# The Effect of the Swimmer’s Trunk Oscillation on Dolphin Kick Performance Using a Computational Method with Multi-Body Motion: A Case Study

**DOI:** 10.3390/ijerph19094969

**Published:** 2022-04-19

**Authors:** Zhiya Chen, Tianzeng Li, Jin Yang, Chuan Zuo

**Affiliations:** 1 School of Industrial Design and Ceramic Art, Foshan University, Foshan 528011, China; zhiya_chen@fosu.edu.cn; 2 Institute of Physical Education, Hunan University, Changsha 410082, China; jinyang@hnu.edu.cn; 3 School of Sports Science, Shanghai University of Sport, Shanghai 200438, China; 1821519002@sus.edu.cn

**Keywords:** dolphin kick, trunk oscillation, hydrodynamic characteristic, swimming velocity, joint moment, vortices

## Abstract

The effect of a specific Chinese swimmer’s trunk oscillation on dolphin kick was investigated in order to optimize competitive swimming movement. Using a numerical simulation method based on multi-body motion, different swimmer’s trunk oscillation during a dolphin kick was analyzed. The simulation was conducted using 3D incompressible Navier–Stokes equations and renormalization group *k-ε* turbulence model, combined with the Volume of Fluid method to capture the water surface. The simulation’s results were evaluated by comparing them with experimental data and with previous studies. The net streamwise forces, mean swimming velocity, and joint moments were also investigated. There was a positive correlation between the mean swimming velocity and the amplitudes of the swimmer’s trunk oscillation, where the Pearson correlation coefficient was 0.986 and the selected model was statistically significant (*p* < 0.05). In addition, as the mean swimming velocity increased from 1.42 m/s in Variant 1 to 2 m/s in Variant 5, the maximum positive moments of joints increased by about 24.7% for the ankles, 27.4% for the knees, −3.9% for the hips, and 5.8% for the upper waist, whereas the maximum negative moments of joints increased by about 64.5% for the ankles, 28.1% for the knees, 23.1% for the hips, and 10.1% for the upper waist. The relationship between the trunk oscillation and the vortices was also investigated. Therefore, it is recommended that swimmers should try to increase the amplitudes of trunk oscillation to increase their swimming velocity. In order to achieve this goal, swimmers should increase strength training for the ankles, knees, and upper waist during the upkick. Moreover, extra strength training is warranted for the ankles, knees, hips, and upper waist during the downkick.

## 1. Introduction

Competitive swimming in the swimming pool, as one of the swimming events, is a competition event based on the velocity of the swimmers. It includes the techniques of starting, swimming on the way, turning and touching the wall at the end, as well as the four styles of freestyle (crawl), backstroke, breaststroke, and butterfly and the medley composed of these four styles. The performance of swimmers depends on the thrust and drag force generated by the interaction between swimmers and the water. The key to improving their performance is to increase the thrust force and to reduce the drag force [1]. Ungerechts [2] emphasized that swimmers employed an undulatory form of movement in water to provide the thrust forces and reduce the drag force. Exploring the problem of the hydrodynamic of swimmers is the main way to optimize swimming movements and to improve training [3].

According to the regulations of the Fédération Internationale de Natation (FINA), the longest underwater swimming stage after the start and turns is 15 m, occupying 30% of the pool length of 50 m. After starting and turning, swimmers generally start dolphin kicks for freestyle, backstroke, and butterfly swimming, and only one dolphin kick for breaststroke. Therefore, the dolphin kick, as the main technique of underwater swimming, has a significant impact on swimming performance [4]. In a dolphin kick, the swimmer’s body swings up and down like a dolphin. According to Atkison et al. [5], each dolphin kick can be divided into two kick phases: downkick and upkick. A downkick started from the highest point of feet to the lowest point, and an upkick started from the lowest point to the highest point. Considering swimmers’ body posture, amplitude, frequency, strength, and shape, a high-efficiency dolphin kick requires swimmers to find a balance between reducing drag force, increasing thrust force and saving energy [6]. In recent years, many studies have focused on the hydrodynamic performance of dolphin kicks [4,7,8,9,10].

Jensen and McIlwain [11] estimated the joint reaction forces involved in a dolphin kick, where the lower limbs of two international competitive swimmers were modeled based on experimental data collected by a point-cloud-data digital analyzer. Their results showed that female swimmers benefitted more from the dolphin kick than male swimmers. Arellano et al. [12] used an underwater camera system to capture the underwater swimming motion. Their results showed that the kick amplitude of international swimmers was greater than that of national swimmers, which led to higher horizontal velocity. Lyttle et al. [13] used a quasi-steady-state simulation method to simulate the dolphin kick. Their results showed that most of the thrust force in the dolphin kick was generated by the legs and the kick efficiency was proportional to the amplitude of dolphin kick. However, Sugimoto et al. [14] used a swimming human model to determine the thrust mechanism and found that most of the thrust force was generated by the feet. In these contradictory studies, the flow field instability of the moving body was ignored, which may be the reason for these different results. Therefore, the interaction between the unsteady flow field and the moving swimmer is the key problem.

Recently, unsteady simulations have been used to study the dolphin kick [4,6,7,10,15,16]. Loebbecke et al. [4,7] and Hochstein et al. [15] showed that most of the thrust force was generated by the feet during kicking. Cohen et al. [6] used the smoothed particle hydrodynamics method to establish a swimming numerical model, which could deal with the complex deformation of swimming. Their results suggest that swimmers’ net thrust force was relatively insensitive to ankle flexibility but strongly dependent on kick frequency. Atkison et al. [5] used underwater cameras to capture underwater swimming movements of 15 international-level male university swimmers, and established a two-dimensional swimmer model. Their results showed that most of the thrust force was generated during the downkicks. Willems et al. [8] conducted trials with 26 high-level swimmers. By using the visual analogue scoring method, it was found that the performance of dolphin kick could be improved through ankle muscle strength training and ankle flexibility training. Yamakawa et al. [17] used a motion capture system to capture the dolphin kick movements of eight males, and established a three-dimensional motion analysis model of hip and knee joints. Their results showed that a positive effect on improving the performance of the dolphin kick was achieved by increasing the extension of knee and accelerating the speed of external rotation of hip and the speed of knee flexion. In addition, Yamakawa et al. [10] optimized maximum knee angle of the dolphin kick using the unstructured moving mesh finite volume method. Their results confirmed that the maximum angle of the knees was one of the factors that have the greatest impact on thrust.

The thrust force of the dolphin kick is determined by the motions of various joints [10], including not only lower limb joints (hips, knee, and ankles) but also the swimmer’s trunk. Compared with the swimmer’s lower limb movement, the trunk movement is also worth investigating. Arellano [18] found that swimmers with smaller oscillations of the trunk had better performance. The appropriate amplitudes and phases of trunk, especially bending at the chest, was important to achieve the maximum propulsive efficiency [19]. Although some studies have found a direct correlation between trunk oscillation and swimming performance, there is a lack of detailed research on the influence of trunk motion on the swimmer’s hydrodynamic characteristics such as drag, thrust force, joint moment, and flow field.

Therefore, the effects of trunk oscillation on the hydrodynamics characteristics of dolphin kick were studied, including swimmer’s net streamwise forces, joint moments, mean swimming velocity, and visualization of the flow field. We hypothesize that a lager oscillation of the swimmer’s trunk has substantial benefits in improving swimming performance.

## 2. Numerical Method

### 2.1. Swimmer Model and Motion Capture

**Figure 1:**
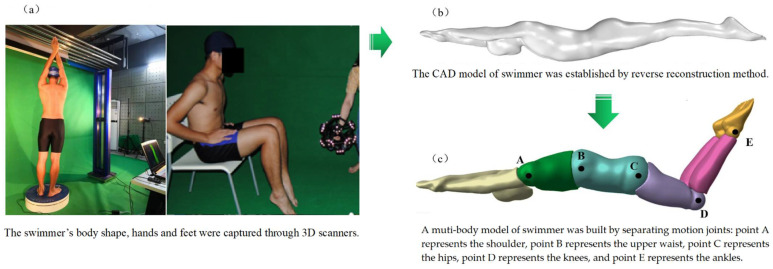
The generation process of the swimmer model. (**a**) The swimmer’s body shape, hands, legs, and feet were captured through 3D scanners; (**b**) The CAD model of swimmer was established by reverse reconstruction method; (**c**) A muti-body model of swimmer was built by separating motion joints: point A represents the shoulder, point B represents the upper waist, point C represents the hips, point D represents the knees, and point E represents the ankles.

Through cooperation with the school of physical education of Hunan University, an elite international swimmer was invited to take a swimming test. The swimmer had participated in the Olympic Games, FINA Swimming World Cup, and Asian Swimming Championships. Moreover, the tag of the swimmer on the FINA website is H. The swimmer weighed 81 kg, and the ratio of his chest circumference, waist circumference, and hip circumference was 1.17:1:1.16. Firstly, a three-dimensional body scanner (ZBOT SCAN-1X) was used to sample the three-dimensional model of the swimmer’s streamline standing posture. This is because the scanning instrument requires people to stand on the rotating disk to scan the whole body. In addition, it is difficult for swimmers to maintain standard swimming posture for a long time when they lie down. In fact, before swimming training, swimmers will practice their swimming posture on the shore in a standing posture. Therefore, the standing posture can ensure the standard of the swimming posture. At the same time, the standing posture will mainly lead to the non-standard posture of hands, legs, and feet, so a hand-held scanner (CYScan 775) was used to scan the action posture of them. Then, the collected 3D point-cloud data were processed using the reverse reconstruction method to generate swimmer’s full-size computer-aided-design (CAD) model. A Kistler KiSwim swimming monitoring system (including five high-speed cameras) was used to capture the underwater movement of swimmers. The movement of the swimmer was assumed to be a coupled movement system of multiple independent limbs (upper limbs, chest, abdomen, thighs, shanks, and feet). The generation process of the swimmer model is shown in Figure 1. More details of the swimming model and motion capture can be found in our previous research [20].

### 2.2. Governing Equations

In the simulation in this study, the Reynolds-averaged Navier–Stokes equations (Equation (1)) were introduced for the incompressible viscous fluid. The renormalization group *k-ε* turbulence model (Equation (2)) was chosen for this turbulence problem with a high Reynolds number. In addition, the Volume of Fluid method (Equation (3)) was chosen for the wave-making behavior on water surface [21]. The equations were as follows:

(1) $$\left\{ \begin{array}{l} \frac{\partial }{\partial t_{i}}\left(u_{i}\right)=0\\ \frac{\partial }{\partial t_{i}}{\left(u_{i}\right)}_{1}+\frac{\partial }{\partial x_{j}}\left(u_{i}u_{j}\right)=-\frac{1}{\rho }\left(\frac{\partial p}{\partial x_{i}}\right)+g_{i}+\frac{\partial }{\partial x_{j}}\left[v\left(\frac{\partial u_{i}}{\partial x_{j}}+\frac{\partial u_{j}}{\partial x_{i}}\right)-\frac{2}{3}\delta_{ij}\frac{\partial u_{l}}{\partial x_{l}}\right]+\frac{\partial }{\partial x_{j}}\left(-\bar{u_{i}^{'}u_{j}^{'}}\right) \end{array}\right.,$$

(2) $$\left\{ \begin{array}{l} \frac{\partial }{\partial t}(\rho k)+\frac{\partial }{\partial x_{i}}(\rho ku_{i})=\frac{\partial }{\partial x_{j}}(\alpha_{k}\mu_{eff}\frac{\partial k}{\partial x_{j}})+G_{k}+G_{b}-\rho \varepsilon +S_{k}\\ \frac{\partial }{\partial t}(\rho \varepsilon )+\frac{\partial }{\partial x_{i}}(\varepsilon u_{i})=\frac{\partial }{\partial x_{j}}(\alpha_{\varepsilon }\mu_{eff}\frac{\partial \varepsilon }{\partial x_{j}})+G_{1\varepsilon }\frac{\varepsilon }{k}(G_{k}+C_{3\varepsilon }G_{b})-C_{2\varepsilon }\rho \frac{\varepsilon^{2}}{k}-R_{\varepsilon }+S_{\varepsilon } \end{array}\right.,$$

(3) $$\left\{ \begin{array}{l} \frac{\partial F_{q}}{\partial t}+\frac{\partial }{\partial x_{i}}(F_{q}u_{i})=0\;(q=1,2)\\ F_{1}+F_{2}=1 \end{array}\right.,$$

where *ρ*, *p*, *k*, *ε*, and *v* are the fluid density, pressure, turbulent kinetic energy, turbulent dissipation rate, and kinematic viscosity, respectively; *u_i_*, *u_j_*, *g_i_*, and $-\bar{u_{i}^{'}u_{j}^{'}}$ are the components of the velocity vector, gravitational acceleration, and Reynolds stresses, respectively; *G_k_* and *G_b_* are the turbulent kinetic energy; *μ_eff_ = μ + μ_t_* and *μ_t_ = ρC_μ_k*^2^*/ε* are the viscosity of water; *R_ε_*_,_ *S_k_*, and *S_ε_* are the terms on *k* and *ε*; *C*_1*ε*_, *C*_2*ε*_, *C*_3*ε*_, *C_μ_*, *α_k_*, and *α_ε_* are constants defined by experience; and *F_q_* denotes the volume fraction of the fluid of the *q-*th phase in the mesh cell.

The dynamic mesh method was employed to simulated the swimming movement. This method can adjust the mesh at any time to adapt to different motions. The equation is as follows:

(4) $$\frac{d}{dt}\int_{V}\rho \phi dV+\int_{\partial V}\rho \phi (\vec{u}-{\vec{u}}_{g})\cdot d\vec{A}=\int_{\partial V}\Gamma \nabla \phi \cdot d\vec{A}+\int_{\partial V}S_{\phi }dV,$$

where $\vec{u},\;\;{\vec{u}}_{g}$, and *Γ* are the flow velocity vector, mesh velocity, and diffusion coefficient, respectively, and $S_{\phi }$ is the source term of general scalar *ϕ*.

### 2.3. Motion Control

In this research, the motion equations of the dolphin dick were derived from the Denavit–Hartenberg modeling method [22]. More details on the motion control can be found in our previous research [20].

In this study, the swimming velocity was calculated according to Newton’s second law in the horizontal direction. The equation is as follows:

(5) $$v_{t}=v_{t-\Delta t}+\left(F/m\right)\Delta t,$$

where $v_{t}$, *F*, and *m* are the swimming velocity, force of the current time, and the mass of the swimmer, respectively, and $v_{t-\Delta t}$ is the swimming velocity of the previous time.

**Figure 2:**
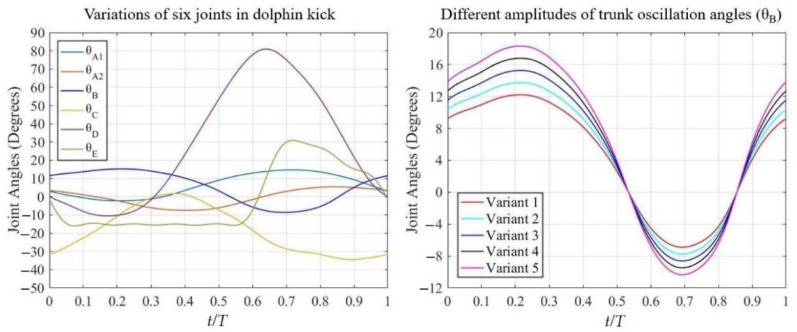
Variations of six joint angles in a dolphin kick cycle and five amplitudes of trunk oscillation angles.

A Kistler KiSwim swimming monitoring system was used to collect the joint movement of the shoulder ($\theta_{A1}$ and $\theta_{A2}$), upper waist ($\theta_{B}$), hips ($\theta_{C}$), knees ($\theta_{D}$), and ankles ($\theta_{E}$), and the measurements were made in Beijing, China. In order to ensure accuracy, these data were collected at least three times to obtain the motion trajectory in the video footage. In addition, the motion trajectory of the numerical simulation was verified through the video images of the swimmer [20]. The variations of six joint angles in a dolphin kick cycle and five different variations of trunk oscillation angles were shown in Figure 2. The trunk oscillation corresponded to the amplitude of joint angles ($\theta_{B}$).

### 2.4. Computational Domain and Numerical Simulation

**Figure 3:**
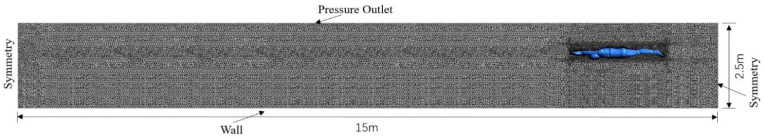
Computational domain and boundary conditions.

In this study, the numerical flume was 15.0 m in length, 2.0 m in width, and 2.5 m in depth, where the water depth was set to 2 m. According to the experiment, the swimmer model was placed 0.45 m below the water surface. Two sides were set as symmetric boundary conditions. The top of the flume was set as the pressure outlet boundary condition. The bottom of the flume and the surface of the swimmer were set as the no-sliding-wall condition. The mesh consisted of tetrahedral grids, and the mesh density around the swimmer was increased (see Figure 3). The Reynolds number was approximately 3.6 × 10^6^ based on the swimmer’s cross section. According to our previous study [16], the minimum grid on the swimmer model was set to 0.005 m, and the total y+ was between 32 and 160, which met the requirements of the simulation model. ANSYS FLUENT V19.0 was used to determine the governing equations. More details about the case setup can be found in our previous research [20].

SPSS Statistics 19.0 (IBM, Armonk, NY, USA) was used to analyze the collected data, and the Pearson correlation coefficient was applied to evaluate the relation of trunk oscillation and the swimming velocity. In addition, the results are considered significant at *p* < 0.05.

## 3. Results and Discussion

### 3.1. Validation of the Velocity

The swimming motion and velocity in the dolphin kick stage were extracted by analyzing the video footage. After statistical analysis, the cycle of a dolphin kick was found to be about 0.48 s, and the average velocity of the swimmer was about 1.8 m·s^−1^. In addition, the average water depth of the swimmer during the dolphin kick was about 0.45 m. The swimmer’s motions were carefully simulated using the user-defined-function code within the ANSYS software, and these motions were visually matched each frame. The velocity of the center under the current numerical simulation was very close to the velocity of the swimmer. More details about motion validation and velocity validation can be found in our previous research [20].

**Figure 4:**
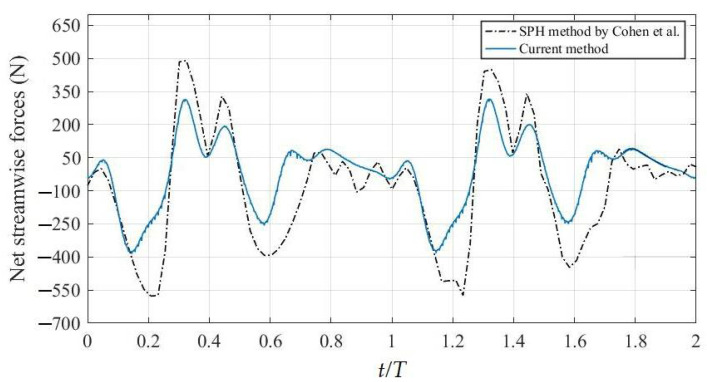
Comparison between the calculated results of this method and the SPH method (Cohen et al. [6]).

Additionally, the net streamwise forces of the swimmer were verified using the data from the smoothed particle hydrodynamics (SPH) method of Cohen et al. [6], as shown in Figure 4. The model movements of Cohen et al. [6] were employed to the current model in our study. The trend of the force curve calculated by the current method was consistent with that of Cohen et al. [6], but the peak values of the propulsive force and drag were smaller than the latter. The differences may correspond to the different swimmer model used in this study. Therefore, the simulation method proposed in this study is feasible.

### 3.2. Net Streamwise Forces

**Figure 5:**
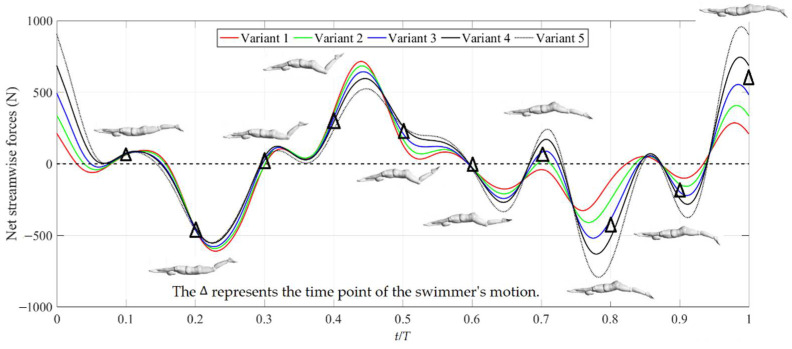
Net streamwise forces of a swimmer in a cycle under different trunk oscillations.

The net streamwise forces of swimmer in a cycle under different trunk oscillations are presented in Figure 5. In Figure 5, the positive force and negative force correspond to the net thrust force and net drag force, respectively. Not only would the downkick produce a lot of thrust force, so would the upkick, which was consistent with the results of Atkison et al. [5] and Shimojo et al. [23]. The streamwise force under all different trunk oscillations was expressed as the drag force when the legs begin to contract upward (*t* ≈ 0.08–0.3 T). The maximum drag (*t* ≈ 0.23 T) corresponded to the maximum frontal area of the swimmer and the reverse movement between legs and the water flow. The streamwise force under all different trunk oscillations was expressed as the thrust force when the legs were at the end of the upkick and the beginning of the downkick (*t* ≈ 0.3–0.6 T). This corresponds to the minimum frontal areas and the same direction movement between the legs and the water flow. In the process of the downkick (*t* ≈ 0.6–0.94 T), the streamwise force was mainly drag force. In addition, the maximum thrust occurred when the legs were at the mid-point of the upkick (*t* ≈ 0.94–1.08 T).

Under all different trunk oscillations, the net streamwise forces were consistent within *t* ≈ 0.08–0.4 T. This corresponded to the same frontal area of the swimmer. At *t* ≈ 0.43 T, the local maximum thrust force was inversely proportional to the amplitude of the trunk oscillations. This corresponded to the higher shape drag due to the higher frontal area of the swimmer. From 0.5 T to 1.08 T, the net streamwise forces were proportional to the amplitude of the trunk oscillations. At *t* ≈ 0.78 T, the drag force in Variant 5 was at a maximum, which corresponded to the maximum frontal area of the swimmer due to the maximum downkick. At *t* ≈ 0.98 T, the thrust force in Variant 5 was also at a maximum, which corresponded to the minimum frontal area of swimmer and a higher kick speed.

**Figure 6:**
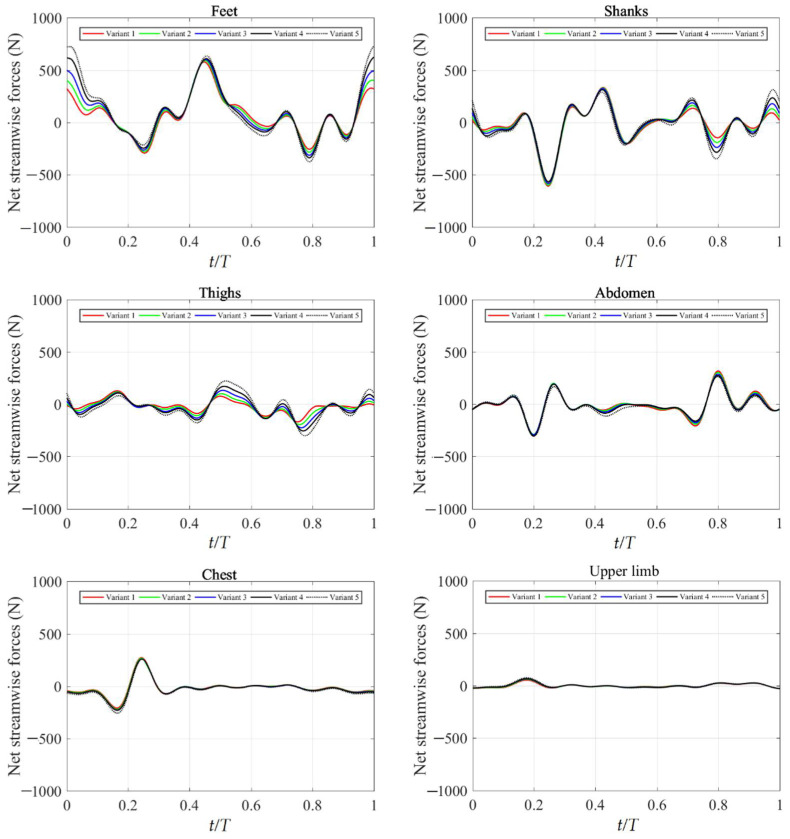
Net streamwise forces of swimmer’s limbs in a cycle under different trunk oscillations.

The net streamwise forces of the swimmer’s limbs in a cycle under different trunk oscillations are presented in Figure 6. The different curves had similar force profiles with no phase differences. Most of the thrust force was generated by the feet, which was consistent with the result of Sugimoto et al. [14]. The higher amplitude of the trunk oscillation, the higher thrust forces generated by the feet. Additionally, most of the drag force was generated by the shanks. The shanks’ net streamwise force of Variant 5 had higher peaks and lower troughs in the range of 0.65–1.0 T. Moreover, the thighs’ net streamwise force for Variant 5 had higher peaks and lower troughs in the range of 0.45–1.0 T. On the contrary, the net streamwise forces were basically the same under different trunk oscillations for the abdomen, chest, and upper limb. The integral of force over time is the impulse. Thus, the impulses of thighs, abdomen, chest, and upper limb were almost zero in a cycle. This corresponded to the relatively small movements of these limbs. These simulation results suggest that swimmers should focus on the movement and training of the feet and shanks, which were the main factors that improve the performance of the dolphin kick.

### 3.3. The Swimming Velocity

**Figure 7:**
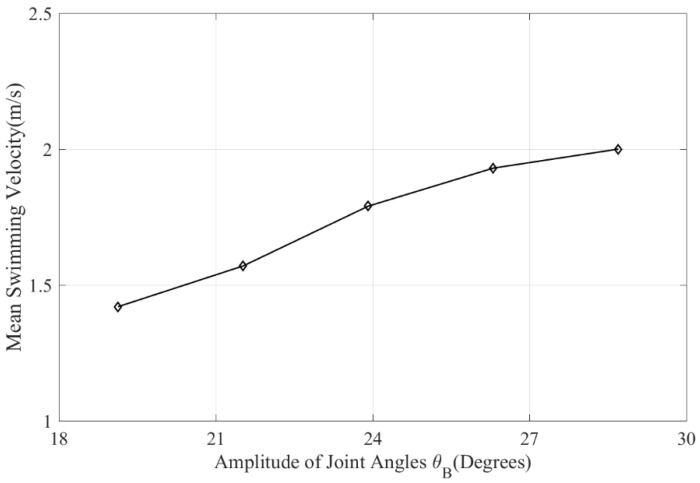
Mean swimming velocity of swimmer under different trunk oscillations.

The mean swimming velocity of swimmer under different trunk oscillations was presented in Figure 7. The trunk oscillation corresponded to the amplitude of joint angles ($\theta_{B}$). This shows that the larger amplitude of joint angles ($\theta_{B}$), the larger mean swimming velocity the swimmer achieved. This corresponds to the net streamwise force in Figure 5 and Figure 6. These variables were positively correlated. The mean swimming velocity of different trunk oscillations increased proportionally with the amplitude of the joint angles ($\theta_{B}$).

The mean swimming velocity of the swimmer under different trunk oscillations was shown in Table 1. The Pearson correlation coefficient of mean swimming velocity and amplitude of joint angles ($\theta_{B}$) is 0.986, and the selected model was statistically significant (*p* < 0.05). Regardless of the energy consumption, a larger kick amplitude could lead to a faster swimming velocity [6], which was also confirmed in the present study. However, maintaining a large kick amplitude for a long time requires high physical fitness for swimmers, especially for the long-distance swimming events. Therefore, the joint moments were also investigated to guide the swimmer’s training.

### 3.4. Joint Moments

**Figure 8:**
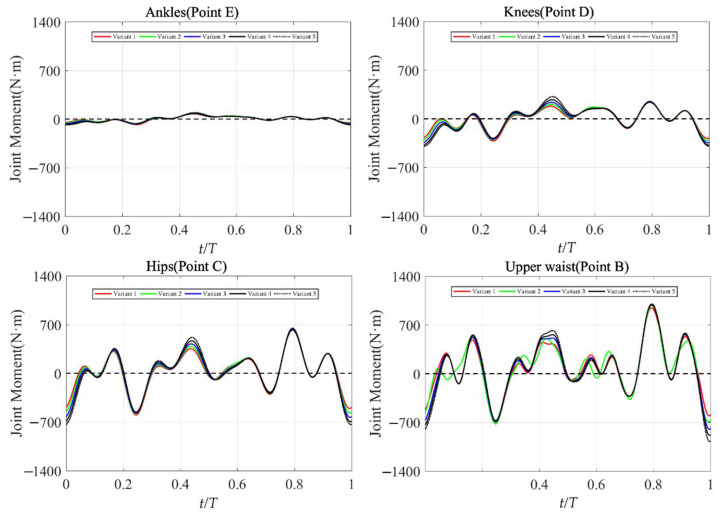
Joint moments under different trunk oscillations.

The performance of the dolphin kick depended on the movement of the limb joints (ankles, knees, hips, and upper waist). As the trunk oscillation changed, the moment of these joints changed, which required swimmers to adjust the training of their corresponding joints. The joint moments under different trunk oscillations are presented in Figure 8. The joint moment of the upper waist was the largest, which was consistent with the opinions of the swimmer. As the amplitude of joint angles ($\theta_{B}$) increased, the maximum positive and negative moments of different joints were also increased. Most of the positive moment was generated during the downkick, whereas most of the negative moment was generated during the upkick. Table 2 presents the maximum positive and negative moments of different joints under different trunk oscillations. For the joint moments of the ankles and the upper waist, the absolute value of the maximum positive moment was larger than that of maximum negative moment. These corresponded to the motion characteristics of these joints; that is, they were responsible for extending the lower limbs during the downkick. Instead, the absolute value of the maximum positive moment was larger than that of the maximum negative moment for the knees. This corresponded to the fact that the knees were responsible for retracting the leg during the upkick. For the hips, as the amplitude of joint angles ($\theta_{B}$) increased, the maximum negative moment increased, whereas the maximum positive moment slightly decreased. This may correspond to the fact that the hips were mainly responsible for kicking the thighs downward. Due to the physiological limitations of the hips, the action of recovering the thighs does not change much. As the mean swimming velocity increased from 1.42 m/s in Variant 1 to 2 m/s in Variant 5, the maximum positive moments of the joints increased by about 24.7% for the ankles, 27.4% for the knees, −3.9% for the hips, and 5.8% for the upper waist, whereas the maximum negative moments of the joints increased about 64.5% for the ankles, 28.1% for the knees, 23.1% for the hips, and 10.1% for the upper waist. As reported by Cohen et al. [6] and Willems et al. [8], the propulsion efficiency was affected by the ankle flexibility and muscle strength, which was also confirmed in this study. The swimmers could benefit from ankle strengthening training [8].

Thus, for the swimmer of this study, the result suggests that he should try to increase the amplitudes of trunk oscillation to increase swimming velocity. However, higher amplitude of trunk oscillation requires more muscle and joint strength, especially for the ankles. For the ankles, the swimmer needed much more strength training for the upkick than for the downkick. For the knees, a same intensity of training was required for the downkick and upkick. For the hips, the joint moment indicated that the swimmer needed more strength training for the upkick, but did not need extra strength training for the downkick. For the upper waist, an appropriate amount of strength training was required based on the existing training.

### 3.5. Visualization of the Flow Field

**Figure 9:**
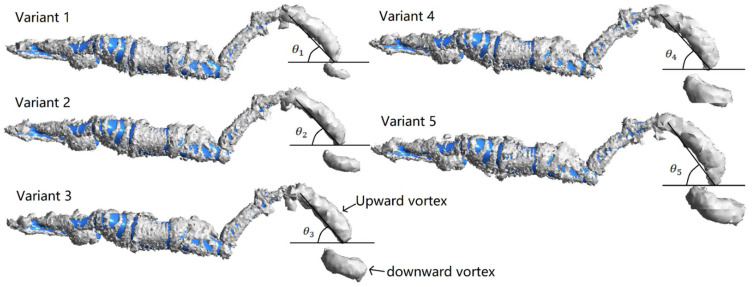
The influence of different trunk oscillations on the vortex structures near the feet at 0.43 T.

The vortex of the flow field is a characteristic of the complex unsteady flow. During the dolphin kick, both the downkick and the upkicks alternately generate vortex structures [6]. Figure 9 shows the influence of different trunk oscillations on the vortex structures near the feet at 0.43 T. As reported by our previous study [20], a strong downward vortex was generated at the end of the upkick, and a strong upward vortex was generated at the end of the downkick. Meanwhile, a strong thrust force occurred. It was clearly observed that the larger the trunk oscillations, the larger the sizes of the vortex structures.

The net streamwise force was deeply involved with the vortex rings generated by the swimmer’s motions. In fact, when the water was pushed backward and formed a vortex, the swimmers achieved forward thrust due to backflushing. Yamakawa et al. [10] reported that the higher the average thrust velocity, the higher the vortex angle changes, and the higher the displacement of the x-direction vortex increases. As shown in Table 3, as the amplitude of the trunk oscillation increased, the angle of the upward vortex increased, as well as the x-direction displacement of upward and downward vortices. Furthermore, Variant 5 has a maximum angle and x-direction displacement of vortices, which corresponded to the maximum mean swimming velocity. Therefore, the behavior of the vortex generated by the feet may be related to effective dolphin kicks, which are worth to study further to explore its mechanical mechanism.

### 3.6. Discussion

The aim of this paper is to study the effect of swimmer’s trunk oscillation on dolphin kick hydrodynamics using a numerical simulation method based on multi-body motion. The results of calculations supported the hypothesis that a lager oscillation of the swimmer’s trunk has substantial benefits in improving swimming performance.

In our study, the cycle of a dolphin kick was found to be about 0.48 s (2.08 Hz), and the average velocity of the swimmer was about 1.8 m·s^−1^. Previously, the dolphin kick in Olympic-level male swimmers were analyzed and reported by Loebbecke et al. [4]. They had an average velocity of 1.50 ± 0.29 m·s^−1^, and a kick frequency of 2.25 ± 0.34 Hz. The kinematic results obtained in this study were larger than those reported in previous studies. We note that our subject was known to have a high performance level in short-distance swimming.

Computational fluid dynamics have been used to study the swimming movements in recent decades. However, there are some doubts about the reliability of numerical calculation as there is a lack of verification of the numerical simulation strategy of swimming. The velocity of the center under the current numerical simulation was very close to the velocity of the swimmer, and the details about motion validation and velocity validation can be found in our previous research [20]. As shown in Figure 4, the variation trend of the net streamwise forces curve calculated by the current method was consistent with that calculated by Cohen et al. [6], but the peak and trough values were smaller than the latter. In fact, the greatest drag force of these two methods both appeared at the late stage of the legs’ retracting movements, when the shape drags increased due to the increase of the swimmer’s projected frontal area. In addition, the swimmer’s maximum thrust force occurred in the stage of downkick, when the feet were more conducive to achieving more thrust. Although the model movements are those of Cohen et al. [6], the swimmer model used in this study is different, and the latter is relatively tall, which may be the main reason for the difference in comparison results. Therefore, the simulation method proposed in this study is feasible.

The key feature of the dolphin kick is an undulatory wave motion that is initiated in the upper part of the body and which increases in amplitude as it propagates down towards the lower limbs (Sanders et al. [24]; Loebbecke et al. [4]). Not only would the downkick produce a lot of thrust force, but so would the upkick, which was consistent with the results of Atkison et al. [5] and Shimojo et al. [23]. Regardless of energy consumption, a higher kick efficiency could lead to a faster swimming velocity (Cohen et al. [6]), which was also confirmed in the present study as shown in Figure 5 and Figure 7. In dolphin kicks, the displacements of the feet and body are normal to the coronal or frontal plane and have minimum magnitude at the hands, and increase along the length of the body, reaching a maximum at the toes (Loebbecke et al. [4]). The dolphin kick is performed using not only the lower limb joints (hip, knees, and ankles), but also trunk and upper limbs undulation. As an important bridge connecting the upper and lower limbs, the trunk needs to cooperate with the swing of the upper and lower limbs to maintain the body balance during swimming. Early observations of swimming competitions found that better performers showed smaller oscillations of the trunk and legs during the undulatory cycle (Arellano [18]). In contrast, current calculations suggest that a higher trunk oscillation is more conducive to increased thrust and reduced drag. That is because a greater trunk oscillation will lead to a greater kick amplitude of the motion of the feet, which is the main source of thrust force.

In the previous studies, the forces of each limb in the whole dolphin kick cycle were calculated by swimming human simulation model without solving the flow field (Nakashima [25]; Nakashima [19]; Nakashima et al. [26]). However, swimming human simulation model is limited due to the simplify of the human model and the lack of the mutual interaction of limbs. Takagi et al. [27,28] developed a humanoid robot and pressure distribution measurement to estimate fluid dynamic forces acting on a hand. However, the robot’s swimming velocity was 0.2–0.24 m·s^−1^, which was far less than the velocity of real swimming. In our study, the forces of each limb of real human model could be clearly distinguished. Figure 6 shows that most of the thrust force was generated by the motion of the feet, which was consistent with the result of Sugimoto et al. [14] and Loebbecke et al. [4]. Additionally, most of the drag force was generated by the motion of the shanks. The change of the trunk oscillations not only affects the net streamwise forces of the trunk itself, but also has a greater impact on those of the lower limbs. The higher amplitude of the trunk oscillation, the higher thrust forces generated by the motion of the feet. From 0.65 T to 1.0 T, peaks and troughs of the shanks’ net streamwise force is directly proportional to the trunk oscillations. On the contrary, the net streamwise forces were basically the same under different trunk oscillations for the abdomen, chest, and upper limb. Moreover, the impulses of thighs, abdomen, chest and upper limb were almost zero in a cycle. These meant the motion of these limbs contribute little to swimming velocity. These results were consistent with the feelings of coaches and swimmers.

In the previous study, the swimming human simulation model was used to determine the effect of the maximum joint moments, and the maximum joint moments were fixed as a single constraint condition. Nakashima and Ono [29] used the swimming human simulation model to solve the arm stroke under different maximum joint moment conditions. On the contrary, the joint moments were investigated and determined by the real movements of the dolphin kicks in our study, as showed in Figure 8. The joint moment of the upper waist was the largest, and that of the hips was the second largest, whereas those of the knees and the ankles were relatively small. As the mean swimming velocity increased from 1.42 m/s in Variant 1 to 2 m/s in Variant 5, the maximum positive moments of the joints increased by about 24.7% for the ankles, 27.4% for the knees, −3.9% for the hips, and 5.8% for the upper waist, whereas the maximum negative moments of the joints increased about 64.5% for the ankles, 28.1% for the knees, 23.1% for the hips, and 10.1% for the upper waist. Connaboy et al. [30] used covariance models to assess the dolphin kick of 17 skilled swimmers, and the results showed that the maximal knee joint angular velocity, maximal ankle angular velocity and knee range of movement were the determinants of the variance in maximal swimming velocity. In our study, the joint moment of the ankles and the knees changed the most, which indicated that the ankles and the knees are more sensitive to the trunk oscillation and are highly related to the swimming velocity. Matsuura et al. [31] reported that both the upkick and downkick followed the trunk muscles, and muscles in the lower limb were activated. The trunk movement during the dolphin kick contributed to improvements in both the swimming velocity and propulsion efficiency (Nakashima [19], Cohen et al. [6], and Willems et al. [8]). The joint moment of the upper waist indicated that the trunk movement is one of the key features to active the motion of lower limbs, and also helps to improve the swimming velocity.

The vortex of the flow field is characteristic of the complex unsteady flow. During a dolphin kick, both the upkicks and downkicks alternately generate vortex structures which emanate from the feet (Ungerechts et al. [32]; Cohen et al. [6]) and move away from body in the same direction. The vortex structures near the feet at 0.43 T are shown in Figure 9, as the maximum trust was generated at the same time in Figure 5. It was clearly observed that the larger the trunk oscillations, the larger the sizes of the vortex structures. In previous study, the higher the average thrust velocity, the more the vortex angle changes and the more the displacement of the x-direction vortex increases [10]. As shown in Table 3, as the amplitude of the trunk oscillation increased, the angle of the upward vortex increased, as well as the x-direction displacement of upward and downward vortices. Therefore, the behavior of the vortex generated by the feet are highly related to effective dolphin kicks, which could be an identification mark to the swimming velocity.

### 3.7. Limitations and Prospects

In this study, the swimmer was assumed to move in the horizontal direction and the fluctuation and rotation were ignored to simplified the simulations. These would lead to a deviation between the simulated results and the actual situation. In addition, this study was conducted on a specific Chinese swimmer, so the conclusions might not be applicable to other swimmers but have reference significance. Therefore, the six-degrees-of-freedom motion method should be used and more case studies are needed in future research.

## 4. Conclusions

Based on multi-body motion, the effect of the swimmer’s trunk oscillation on the hydrodynamics of dolphin kicks has been studied with the numerical simulation method. The simulation method was proved to be feasible by the comparison with an experiment and with previous research results. The main conclusions are as follows:

(1) The hydrodynamic characteristics (drag, thrust, mean swimming velocity, joint moments, flow field visualization, etc.) of the dolphin kick were successfully investigated, especially the joint moments. This method may be helpful for swimmers to improve their performance and training.
(2) The maximum drag and thrust force increased as the amplitude of trunk oscillations increased, which led to the increase in mean swimming velocity. It is, therefore, recommended that swimmers should try to achieve a larger amplitude of trunk oscillations to improve swimming performance.
(3) As the amplitude of trunk oscillations increased, the moments of lower limb joints (ankles, knees, hips, and upper waist) also increased. When the mean swimming velocity increased from 1.42 m/s in Variant 1 to 2 m/s in Variant 5, the maximum positive moments of joints increased by about 24.7% for the ankles, 27.4% for the knees, −3.9% for the hips, and 5.8% for the upper waist, whereas the maximum negative moments of joints increased by about 64.5% for the ankles, 28.1% for the knees, 23.1% for the hips, and 10.1% for the upper waist. These suggest that swimmers should increase strength training for their ankles, knees, and upper waist during the upkick. Moreover, extra strength training is recommended for the ankles, knees, hips, and upper waist during the downkick.
(4) As the mean swimming velocity increased with the lager trunk oscillation, the angle of the upward vortex increased, as well as the x-direction displacement of the upward and downward vortices. Therefore, the behavior of the vortex generated by the feet may be related to effective dolphin kicks, which are worth studying further to explore its mechanical mechanism.

## Figures and Tables

**Table 1:** Mean swimming velocity of the swimmer under different trunk oscillations.

	Variant 1	Variant 2	Variant 3	Variant 4	Variant 5
$\mathrm{Amplitude}\;\mathrm{of}\;\mathrm{Joint}\;\mathrm{Angles}\;\theta_{B}$	19.13°	21.53°	23.92°	26.31°	28.70°
Mean swimming velocity (m/s)	1.42	1.57	1.79	1.93	2.00

**Table 2:** Maximum positive and negative moments of different joints under different trunk oscillations.

Variant Number	Maximum Moment (N · m)	Ankles	Keens	Hips	Upper Waist
Variant 1	positive	74.74	250	647.2	945
	negative	−51.14	−310	−589.2	−886.5
Variant 2	positive	77.8	250	648.1	994
	negative	−57.59	−313.7	−585.9	−884.4
Variant 3	positive	80.89	249.3	647.2	986.8
	negative	−65.66	−346.1	−632.4	−895.6
Variant 4	positive	84.73	274	638	986.9
	negative	−76.2	−378.3	−692	−886.7
Variant 5	positive	93.22	318.5	621.8	1000
	negative	−84.12	−397.2	−725.4	−976.3

**Table 3:** Comparison of angle and the x-direction displacement of vortices.

Variant Number	Angle of Upward Vortex *θ* (°)	X-Direction Displacement of Upward Vortex (m)	X-Direction Displacement of Downward Vortex (m)
Variant 1	44.47	0.4585	0.5310
Variant 2	45.02	0.4990	0.6266
Variant 3	46.09	0.5484	0.6760
Variant 4	47.07	0.5519	0.6325
Variant 5	51.71	0.5802	0.7457

## Data Availability

The data used to support the findings of this study are available from the corresponding author upon request.
